# Assessment and Estimation of Face Detection Performance Based on Deep Learning for Forensic Applications †

**DOI:** 10.3390/s20164491

**Published:** 2020-08-11

**Authors:** Deisy Chaves, Eduardo Fidalgo, Enrique Alegre, Rocío Alaiz-Rodríguez, Francisco Jáñez-Martino, George Azzopardi

**Affiliations:** 1 Department of Electrical, Systems and Automation, Universidad de León, 24007 León, Spain; eduardo.fidalgo@unileon.es (E.F.); enrique.alegre@unileon.es (E.A.); rocio.alaiz@unileon.es (R.A.-R.); fjanm@unileon.es (F.J.-M.); 2 Researcher at INCIBE (Spanish National Cybersecurity Institute), 24005 León, Spain; 3 Bernoulli Institute for Mathematics, Computer Science and Artificial Intelligence, University of Groningen, 9747 AG Groningen, The Netherlands; g.azzopardi@rug.nl

**Keywords:** face detection, CSEM, deep learning, GPU, CPU, Benchmark, regression

## Abstract

Face recognition is a valuable forensic tool for criminal investigators since it certainly helps in identifying individuals in scenarios of criminal activity like fugitives or child sexual abuse. It is, however, a very challenging task as it must be able to handle low-quality images of real world settings and fulfill real time requirements. Deep learning approaches for face detection have proven to be very successful but they require large computation power and processing time. In this work, we evaluate the speed–accuracy tradeoff of three popular deep-learning-based face detectors on the WIDER Face and UFDD data sets in several CPUs and GPUs. We also develop a regression model capable to estimate the performance, both in terms of processing time and accuracy. We expect this to become a very useful tool for the end user in forensic laboratories in order to estimate the performance for different face detection options. Experimental results showed that the best speed–accuracy tradeoff is achieved with images resized to 50% of the original size in GPUs and images resized to 25% of the original size in CPUs. Moreover, performance can be estimated using multiple linear regression models with a Mean Absolute Error (MAE) of 0.113, which is very promising for the forensic field.

## 1. Introduction

Forensic laboratories very often examine digital evidence during a criminal investigation. In particular, the criminal investigation of Child Sexual Exploitation Material (CSEM) shows a growing interest internationally [1]. Advances in technology have increased the use of mobile devices, social media and P2P networks, making it easier for offenders to create and distribute CSEM, something that has become highly prevalent worldwide.

Given this scenario, a manual analysis to identify new CSEM in any seized electronic device (hard drive, desktop, smart phone, and memory stick, among others) becomes absolutely infeasible within the proposed time constraints of most investigations. Not only is it a very time-consuming and expensive task, but it also exposes image analysts to sensitive and disturbing data on a daily basis, which can affect their emotional state and consequently their performance. Hence, the development of fast, automatic and efficient tools for the automated discovery and analysis of images and videos to be implemented in criminal laboratories becomes crucial for the forensic field [2,3].

Although image analysts can easily identify multiple objects in an image with little or no conscious thought, a high-level understanding from digital images or videos can also be achieved using computer vision [4]. The availability of huge amounts of data, hardware resources and machine learning techniques allow to train computers to derive meaningful information from images. Thus, CSEM can be identified with image classification techniques [5], and its content can be explored using object detection methods [6,7,8,9] and object recognition techniques [10,11,12,13].

Our approach to the CSEM detection is based on the combination of face detection, age estimation and pornography detection. In this work, we address the problem of accurate and fast face detection since it plays a key role in automatic CSEM detection systems. These systems aim to help speeding-up the analysis process, extracting from the vast amount of files stored in a given device those that are suspicious to have child sexual abuse content.

Automatic face detection, meaning the localization of regions that contain faces in digital images, is a topic widely studied in the past decades due to its wide range of applications that involve face analysis. Such practical problems include forensic/security applications like video surveillance for criminal activity detection, facial recognition of fugitives and victims of child sexual abuse, among others.

Typically, automatic face detection is the first step towards face-related applications and it is expected to identify faces under arbitrary image conditions. In real-world settings, the face detector should be robust enough to detect faces in low-resolution and low-quality images with occlusions, changes in pose/illumination and distortions, such as out-of-focus blur, noise and low contrast [14,15,16], which are commonly present in CSEM. However, automatic face detection is a very challenging task in these conditions since performance degradation has been observed while testing detectors on low-quality images [15]. There are basically two main approaches to address this problem [14]: those based on hand-crafted descriptors and the ones based on trainable features with deep-learning techniques.

Traditional face detectors are based on hand-crafted features, such as cascade methods or Deformable Parts Model (DPM). For cascade techniques, the work by Viola-Jones [17] with the AdaBoost cascade scheme using Haar features is the most representative approach. After that, many works followed this direction and more features were proposed with a similar structure of the Viola-Jones detector, including SURF [18], HoG [19] and LBP [20]. Another class of face detection methods based on structured models rely on DPM [21] to cope with the intraclass variance. The aforementioned features may have a limited modeling and representational power to deal with difficult detection conditions like low/high illumination, face occlusion, expression and low-quality images.

Nowadays, deep learning approaches are widely applied for face detection as they enable the system to automatically learn representations from raw input images using a Convolutional Neural Network (CNN), achieving a high accuracy under very challenging detection conditions [22,23,24,25,26,27,28,29]. Most of the deep-learning-based face detectors are, however, computationally demanding and may not be suitable for applications that analyze large amounts of data and require real-time performance, such as the CSEM detection systems in forensic tools.

Usually, traditional [17,18,19,20,21] and deep-learning-based face detectors [22,23,24,25,26,27,28,29] are designed to process 2D images, but they have also been extended to analyze 3D data [30,31,32,33]. These approaches take advantage of deep information to be less sensitive to low/high illumination conditions and viewpoint variation in comparison to 2D face detectors. Nevertheless, this is also a drawback, since 3D approaches are computationally more complex than 2D ones. Therefore, we limited this research to 2D face detectors since the application domain of the study is forensic tools, where real-time performance is crucial.

An image resizing strategy, specifically valid for CSEM, has been presented in [34] to improve the speed–performance tradeoff of three deep learning based face detectors. The face detectors evaluated were selected according to their processing time and accuracy performance: Multi-Task Cascade CNN (MTCNN) [22], the Context-Assisted Single Shot Face Detector (often referred to as PyramidBox) [25] and Dual Face Shot Detector (DSFD) [26]. The validation of this strategy was, however, limited to one GTX 1060 GPU, and the results showed that the image resizing strategy can speed-up face detection with a small reduction in accuracy. A posterior work [35] also showed that it is possible to find a good balance between speed and performance with this resizing strategy.

There is a large variety of available Intel CPUs (https://www.intel.co.uk/content/www/uk/en/products/processors/core.html) and Nvidia GPUs in the market like Tesla (https://www.nvidia.com/en-us/data-center/v100), TITAN (https://www.nvidia.com/en-us/deep-learning-ai/products/titan-rtx), GTX (https://www.nvidia.com/en-us/geforce/10-series) or RTX (https://www.nvidia.com/en-us/geforce/20-series) series, with different specifications that might also speed-up face detection. End users, like law enforcement analysts, often face the problem of choosing the most suitable hardware for the analysis of forensic material at hand.

In this paper, we present a comprehensive comparison of the tradeoff between speed and accuracy of face detection methods through the image resizing strategy presented in [34] for a wide variety of hardware architectures. Specifically, we evaluate five Intel CPUs—i5-3450, i7-4790K, i7-8650U, i9-8950HK, and Xeon E5-2630—and seven Nvidia GPUs—Tesla K40, TITAN Xp, GTX 1050, GTX 1060, GTX 1070, RTX 2060, and RTX 2070. We evaluated three representative face detection methods, namely MTCNN, PyramidBox and DSFD, using a set of images chosen from the WIDER Face data set [36] and the Unconstrained Face Detection Data set (UFDD) [37]. The selected images contain less than five people per scene in order to replicate the number of individuals observed in CSEM images. Additionally, we train a model that is able to predict for unseen images, the performance metrics (in terms of accuracy and speed) that the end-user could expect based on the given face detection method, specific hardware, image size and percentage of image resizing. This research work is part of the European project Forensic Against Sexual Exploitation of Children (4NSEEK) and the research lines defined by the Framework agreement between INCIBE (Spanish National Cybersecurity Institute) and the University of León. Conclusions drawn from this study can be used as a face detection benchmark for users of the 4NSEEK tools in order to guide them in the selection of hardware for the analysis and categorization of CSEM.

The rest of the paper is organized as follows. Closely related work to the one addressed in this paper is presented in Section 2. The evaluation methodology proposed in this work is described in Section 3. Experimental evaluation is described in Section 4 and results are shown in Section 5. Finally, we draw conclusions in Section 6.

## 2. Related Work

Both processing time and accuracy are important performance issues for face detectors. The mean Average Precision (mAP) is an appropriate and widely used metric to assess the accuracy of object detectors [38]. Basically, it computes the area under the precision–recall curve obtained by applying several decision thresholds. Research efforts have focused on improving simultaneously both of them.

Recent advances in deep learning methods have contributed to significant performance improvements in a wide range of computer vision applications. They have been particularly successful for face detection problems where modern deep CNN models show a significant accuracy improvement in comparison to traditional approaches based on hand-crafted features [22,23,24,25,26,27,28,29,39,40,41,42,43,44,45]. Consequently, these deep learning methods have become the state-of-the-art for face detection.

The MTCNN [22] method uses custom CNNs to solve simultaneously the problem of face detection and alignment in real-time. It consists of three sub-networks that process the faces from coarse to fine. Compared with traditional methods, it has a better performance and faster detection speed, but it may show a low performance on low-quality images. Robust features obtained with standard CNNs like VGG16 [46] are, therefore, employed to improve face detection in these conditions [23,24,25,26]. In particular, the Single Shot Scale-Invariant Face Detector (S3DF) [24] method increases the recall of small faces by predicting candidate locations of faces on multi-scale feature maps extracted with VGG16.

The Feature Agglomeration Networks for Single Stage Face Detection (FANet) [23] and PyramidBox [25] methods integrate multi-scale feature maps with multi-level semantic information to improve the detection of small faces. Similarly, DSFD [26] aggregates multi-scale and semantic information with enhanced features corresponding to context information to increase face detection accuracy. More recently, AInnoFace [27] uses the RetinaNet detector [47] in addition to several optimization strategies to improve the detection of tiny faces and outperforms most of the state-of-art methods on the WIDER Face data set [36].

Table 1 reports the mAP and the speed values indicated by the reviewed detectors on the WIDER Face data set, which contains images labeled into three detection difficulty categories: ‘Easy’, ‘Medium’ and ‘Hard’ based on the detection rate of the EdgeBox [48].

New face detectors are commonly evaluated in the literature in terms of their accuracy, which is usually quantified by the mAP metric. Their speed is somehow overlooked and rarely reported. Zhang et al. [49] addressed this issue and presented a CNN-based face detector with a good tradeoff between accuracy and speed, considering both CPU and GPU and emphasising the worth of building effective models without being computationally prohibitive. The detection speed is, however, a relevant factor for end-users taking into account (i) the complexity of some of these models, (ii) the effect of the face detection step in the processing time of several applications where it is required to process large amount of data as found in forensic ones, and (iii) the wide offer in the market of CPUs and GPUs that may help to speed up deep-learning-based detectors.

A comparison of the required training time for several deep learning frameworks during the object classification task with various CPUs and GPUs is presented in [50,51], but they lack analysis of the speed at testing/deployment phase. The performance of common image processing algorithms, such as image segmentation, rotation and deblurring, was studied in [52]. That work, however, did not consider more complex tasks and it was limited to a small number of CPUs and GPUs. To the best of our knowledge, there is no study that can be used as a benchmark for face detection performance with several hardware configurations. Thus, we aim to provide one with this work.

## 3. Methodology

**Figure 1:**
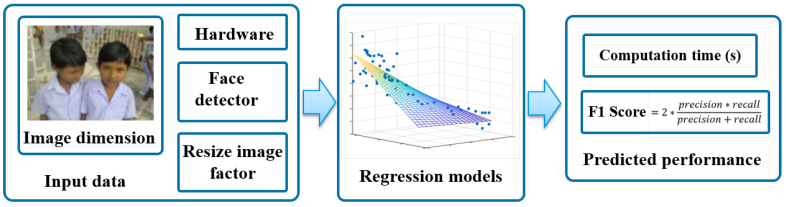
Strategy to predict the face detection performance to an input image.

We aim to provide a guide for end-users to choose the most appropriate hardware for face-related applications, such as face recognition or child detection in CSEM. We address this objective in two ways. First, we present a comparison of the tradeoff between speed and accuracy metrics of face detection through the image resizing strategy described in [34] for several CPUs and GPUs with a small number of subjects per image in order to simulate CSEM. Second, using the collected information regarding the face detection performance, we train a model to predict the behavior of a face detector, in terms of speed and accuracy, in an image based on specific hardware, image size and percentage of image resizing, Figure 1.

### 3.1. Image Data Sets

We evaluate and gather the performance of face detectors, namely computation time and accuracy metrics (mAP and F1 score), by analyzing images from two data sets: WIDER Face [36] and UFDD [37]. Images on the WIDER Face data set contain a large number of real world scenes from 60 events, which are labeled by three levels of difficulty to detect faces: easy, medium and hard (see Table 2). Images in the UFDD data set are labeled into seven categories: rain, snow, haze, blur, high/low illumination, lens distortion, and distractors.

These two data sets were chosen because they consider a wide range of acquisition conditions, including a high degree of variability in illumination, scale, pose and occlusion. Moreover, they allow to evaluate the generalization capability of face detectors since images in the WIDER Face data set have the same acquisition conditions of the data commonly used to train detectors [22,25,26]. On the contrary, images in the UFDD Face data set comprise conditions that are not usually considered in facial images data sets, such as weather degradation, motion and focus blur. Furthermore, analyzing images with a wide range of conditions allows to address different realistic CSEM situations.

In order to try to replicate the usual number of subjects involved in CSEM, only images with less than five people were analyzed. A total of 1994 images with 3358 faces, and resolution between 218×1024 and 1027×1024 pixels were manually chosen from the WIDER Face data set. Moreover, a total of 2222 images with 4214 faces, and resolution between 301×1024 and 1029×1024 pixels were selected from the UFDD data set.

### 3.2. Face Detectors

Following the study in [34], we select three popular face detectors with publicly available implementations for analysing their performance: MTCNN [22,53,54,55], PyramidBox [23,25,56,57] and DSFD [26,56,58].

MTCNN simultaneously applies face detection and face alignment to improve the detection of rotated faces. This method uses three CNNs: the first one obtains candidate regions that may contain faces, the second improves the initial face detection by rejecting false positive candidates and refining face locations, and the third CNN detects facial landmarks. MTCNN overcomes the limitations of other CNN models by considering the diversity of weights and reducing the number of filters and their sizes. MTCNN is the face detector currently integrated in the Evidence Detector software, provided by INCIBE to the two Law Enforcement Agencies that operate in Spain (*Policia y Guardia Civil Española*).

PyramidBox, a context-assisted single shot face detector, combines high-level context semantic features and low-level facial features to predict faces in different scales in a single shot which improved the detection of small faces. In addition, PyramidBox uses feature maps generated at different levels and anchors with an extended VGG16 as backbone and Data-anchor-sampling to increase the diversity of training data.

DSFD extended SSD [59] by integrating feature maps obtained from a VGG16 architecture with enhanced feature maps. It uses a Feature Enhancement Module, which uses information from different levels. Moreover, DSFD introduced a collaborative face sampling and anchor design during augmentation to enhance regressor initialization. This module boosted the semantics of the features and improved the locations of faces in difficult detection conditions.

### 3.3. Resizing Strategy

**Figure 2:**
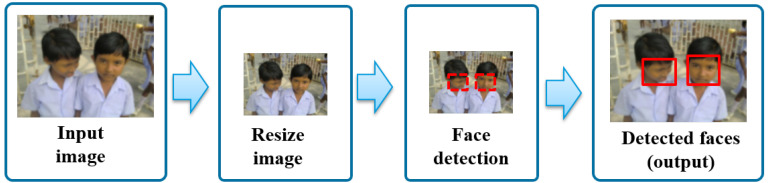
Pipeline of detecting faces after resizing.

In Figure 2 we illustrate the data flow of the resizing strategy that we evaluate. We use it to substantially decrease processing time for face detection in [34]. First, the largest dimension of the image—height or width—is used as reference to reduce the image resolution in a percentage of their original size using bilinear interpolation. This allows us to keep the aspect ratio proportional of the image content including faces. Secondly, bounding boxes corresponding to the face locations are detected on resized images using a deep-learning based method. Finally, detected bounding boxes containing face locations are scaled back to the original image dimensions and returned as output. This step is necessary due to the fact that detected faces would be used in applications where face locations are expected in original image coordinates, such as face recognition, age and gender estimation.

We compare the performance of the selected face detectors on several hardware architectures with four relative sizes—100%, 75%, 50% and 25% of the original dimensions—by following the image resizing strategy described above.

### 3.4. Hardware

We choose a representative group of Intel CPUs—i5-3450, i7-4790K, i7-8650U, i9-8950HK, and Xeon E5-2630—and Nvidia GPUs—Tesla K40, TITAN Xp, GTX 1050, GTX 1060, GTX1070, RTX 2060, and RTX 2070—to ensure an exhaustive evaluation of the selected face detectors. Table 3 and Table 4 summarise the main specification details of the selected CPUs and GPUs.

### 3.5. Prediction of Face Detection Performance Using Regression Models

Face detection is a crucial step in several applications, however the most accurate methods require high computational resources and processing time that may be limited in some domains, such as forensics, where (near) real time performance is expected. Taking into account the use of downsampled images speeds up the face detection stage but reduces the accuracy of the methods, it is desirable to predict the performance of several face detectors with various resized images in a specified hardware. This will enable the end-user to select the best parameters (method and image resolution) for face detection considering the available computational resources.

In this work, we built regression models to predict the face detection performance (computational time and F1 score) using five explanatory variables: the input image size (width and height), the image resized percentage (100%, 75%, 50% and 25% of the original image size), the face detector (MTCNN, PyramidBox, DSFD), and the hardware (specific CPU or GPU). We collected the face detection performance information considering the image data sets, detectors, resizing strategy, and hardware described above.

## 4. Experimental Setup

Experiments were run on a GNU/Linux machine box with Ubuntu 18.04, Cuda 9, and CuNDD 7 to: (i) compare the tradeoff between the accuracy and the speed of publicly available implementations of the face detectors—MTCNN, PymaridBox and DSFD—using as input four relative sizes—100%, 75%, 50% and 25% of the original sizes—in several GPUs and CPUs described in the Section 3, and (ii) evaluate the models built to predict the performance of face detectors in a given image with a specific hardware.

Face detectors were coded with Python 3 (https://www.python.org/) and Tensorflow (https://www.tensorflow.org/). Both of them are commonly used to design, build, and train deep learning models. During the assessment of detectors in CPUs and GPUs, images containing less than five individuals, which is the usual number of subjects observed in CSEM, were processed sequentially. Moreover, in case computers were equipped by GPUs had their GPUs disabled in order to exploit the CPU computational capability during evaluation. Also, the usage of GPU memory was not limited during GPU tests, and it was set to grow as needed by the face detectors.

In order to evaluate the tradeoff between the accuracy and the speed of the face detectors, we assessed the accuracy using the mean Average Precision [38] (mAP) and the F1 score metrics [60]. The mAP combines the precision and recall measures by summarizing the shape of the precision–recall curve. The mAP is defined as the mean precision at a set of eleven equally spaced recall levels computed for a threshold that varies from 0 to 1 in intervals of 0.1. An interpolation of the precision–recall curve is used to reduce the impact of the “wiggles” in the curve, caused by small variations in the ranking of examples. The F1 score is the harmonic mean of the precision and the recall measures considering a threshold of 0.5 against ground truth regions to determine true positive and false positive face detections. Furthermore, we computed an improvement (Impv) measure to compare the performance of the face detectors considering the analyzed input image sizes and hardware. The improvement is defined in Equation (1) as the relative difference between the baseline configuration, *A*, and another one, *B*.

(1) $$Impv=\frac{A-B}{A}\times 100$$

Positive values of Impv indicate that *B* outperforms *A* in terms of the evaluated performance metrics (mAP, F1 score or speed).

Regression models to predict the face detector speed were built considering a set of 461,826 examples with the four explanatory variables described in the Section 3: the input image size, the image resized percentage, the face detector and the type of hardware. We randomly split the data set into 80% for training and the remaining was used for the evaluation of the speed prediction model. Regression models to predict the F1 score metric of face detectors were trained considering only three explanatory variables (the input image size, the image resized percentage, and the face detector) since the detectors have the same F1 score performance regardless of the hardware used for the analysis. In this case, a set of 38,616 examples was used to build the prediction models, which was split into a training set with 80% of the examples and a test set with the remaining 20%.

In both cases, we evaluated the regression models using the Mean Absolute Error (MAE), the Mean Squared Error (MSE), and the Root Mean Squared Error (RMSE) [61]. These measures are defined below for a set of *n* samples where *y* is the ground truth value and $\hat{y}$ is the predicted value.

(2) $$MAE=\sum_{i=1}^{n}\frac{\left|y_{i}-{\hat{y}}_{i}\right|}{n}$$

(3) $$MSE=\sum_{i=1}^{n}\frac{{\left(y_{i}-{\hat{y}}_{i}\right)}^{2}}{n}$$

(4) $$RMSE=\sqrt{\sum_{i=1}^{n}\frac{{\left(y_{i}-{\hat{y}}_{i}\right)}^{2}}{n}}$$

The MAE corresponds to the average of the absolute errors of the prediction model and indicates how close are the predicted values to the ground truth. The MSE measures the average of the square errors of the regression model. The RMSE corresponds to the standard deviation of the errors of the model. The closer MAE, RMSE and MSE are to zero, the better the regression models perform.

## 5. Experimental Results

### 5.1. Speed–Accuracy Tradeoff Analysis

Evaluation metrics computed for face detectors (processing time, F1 score, mAP, precision–recall curves, and Impv) were grouped by the image data set (WIDER Face and UFDD) and discussed in the next two sections.

#### 5.1.1. Results on the WIDER Face Data Set

**Figure 3:**
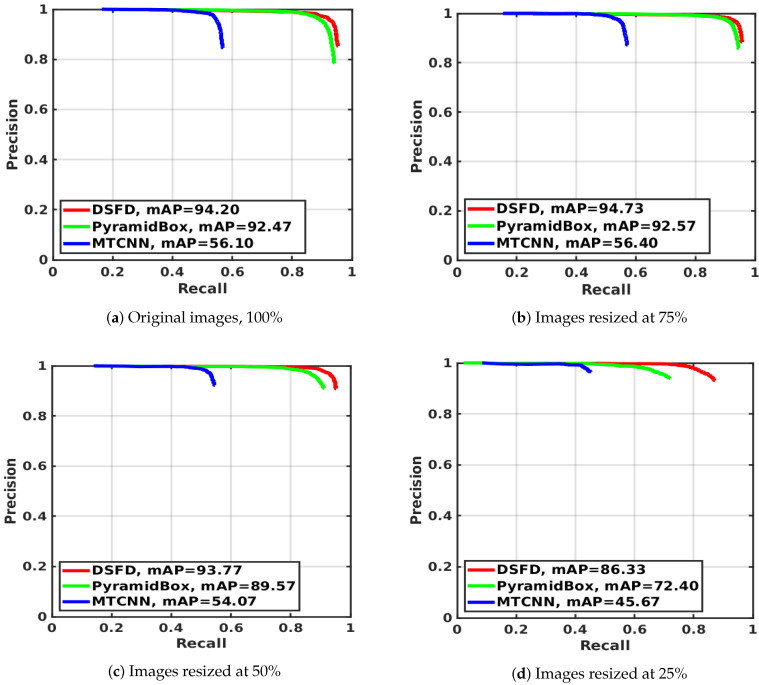
Precision-Recall curves on WIDER Face data set for MTCNN, PyramidBox and DSFD face detection methods using four different image resolutions.

**Figure 4:**
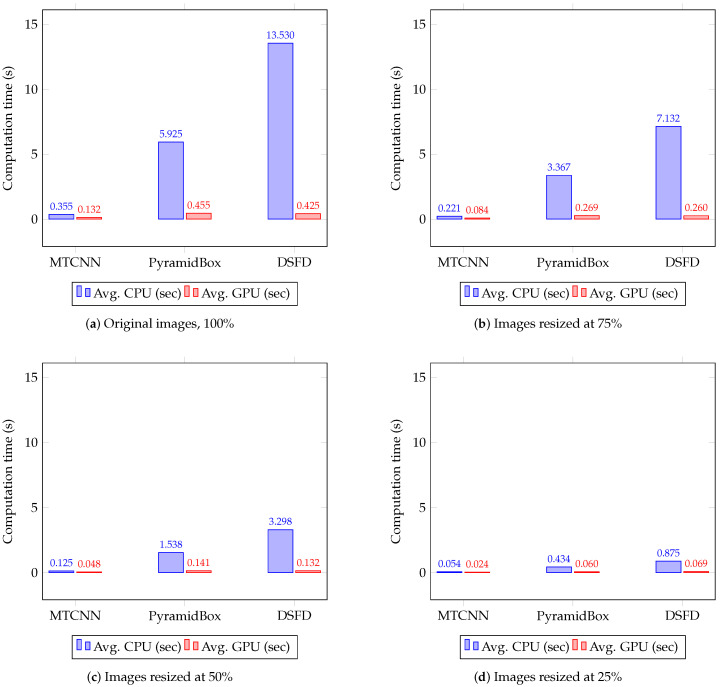
Average CPU and GPU computation time(s) on the WIDER Face data set for MTCNN, PyramidBox and DSFD face detection methods using four different image resolutions.

Table 5 shows the mAP, the F1 score and the face detection speed (in seconds) computed for the evaluated hardware and image sizes. Table 6 presents the Impv of the mAP, the F1 score and the processing time by comparing the detectors’ performance with three relative sizes—75%, 50% and 25%—against the results with original images. Figure 3 presents the precision–recall curves for the evaluated detectors, and Figure 4 exhibits the average computation time on CPUs and GPUs. In both cases, results are grouped by the evaluated image sizes.

As can be seen in Table 5, Figure 3 and Figure 4, MTCNN is the fastest and the least accurate detector, while DSFD is the slowest and the most accurate method. MTCNN has mAP values between 45.67% and 56.40%, F1 score range between 0.392 and 0.505 and a maximum processing time of 0.573 sec in CPUs and 0.200 sec in GPUs. DSFD presents mAP values between 86.33% and 94.73%, F1 score values between 0.743 and 0.917 and maximum detection time of 18.211 sec in CPUs and 0.829 sec in GPUs. In most cases, the use of GPUs significantly speed-up the detection in comparison to CPUs. The results show that GPU detection times outperformed the CPU ones (improvement between 55.51% and 96.86%). This percentage of Impv is related to the complexity of the methods. In general, complex detectors, such as PyramidBox and DSFD, have a large speed-up by using GPUs. Hence, using GPUs, MTCNN presented a speed improvement Impv between 55.51% and 62.70%, while DSFD speed increased between 92.14% and 96.86%.

Furthermore, the use of resized images improves detection speed in comparison with the analysis of full size images (see Table 6). A large resized image percentage leads to a significant processing time decrease. Therefore, the maximum speed-up, in both CPUs and GPUs, is observed with images resized to 25% of the original size. In this case, the fastest face detection is achieved using GPUs with an average (Avg.) Impv in speed of 81.81% for MTCNN, 85.45% for PyramidBox and 82.92% for DSFD. Although face detection is performed faster in GPUs, the use of resized images allow a larger speed-up during CPU analysis with an Avg. Impv in detection times of 85.24% for MTCNN, 92.53% for PyramidBox and 93.46% for DSFD.

However, as expected, the resizing strategy leads to a reduction of accuracy metrics (the mAP and the F1 score), related to the modified size of the images. In particular, large resized image percentages led to higher decreases in mAP and F1 scores in comparison to the performance obtained using full size images. Thus, the maximum mAP and F1 score reduction are observed with images resized to 25% with a drop for MTCNN in mAP of −18.60% and F1 score of −22.35%, a reduction for PyramidBox in mAP of −21.70% and F1 score of −35.72%, and a decrease for DSFD in mAP of −8.35% and F1 score of −18.91%. The best accuracy performance is achieved with images resized to 75% where the mAP slightly improved in comparison to the mAP values obtained with full size images. Thus, it ranges from 0.11% for PyramidBox to 0.53% for MTCNN and 0.57% for DSFD. Moreover, DSFD performed better than MTCNN and PyramidBox with resized images.

**Figure 5:**
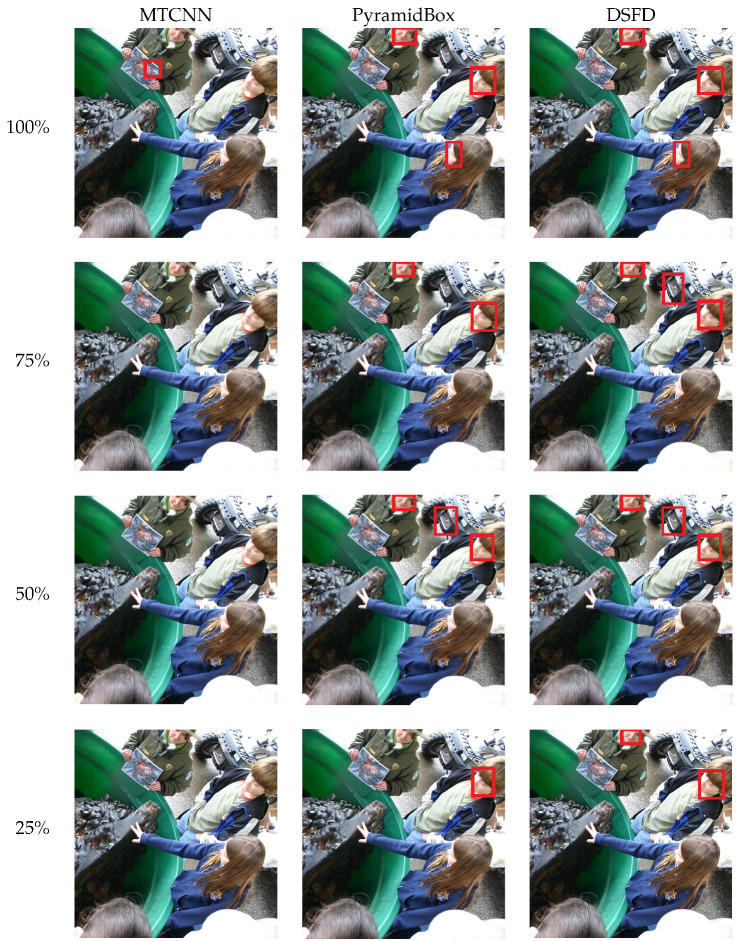
Detected faces using the MTCNN, PyramidBox and DSFD methods with four image resolutions.

Figure 5 illustrates the face detection results on images with subjects in a side-view position (difficult pose). In these conditions, MTCNN does not detect any face from the original or resized images. While the most robust face detectors—DSFD and PyramidBox—detect faces in all the evaluated cases. The precise localization of the bounding boxes around the detected faces are affected by the dimensions of the concerned image.

The best speed–accuracy tradeoff is obtained on the WIDER Face data set using DSFD and GPUs with images reduced to 50% of the original size or CPUs with images reduced to 25% of the full size (see Table 5 and Figure 4). In the case of the GPU analysis, the accuracy—Avg. mAP of 93.77% and Avg. F1 score of 0.883—is improved and the computation time is not affected—0.132 sec—in comparison to the results observed with MTCNN and full size images—Avg. mAP of 56.10%, Avg. F1 score of 0.505, and processing time of 0.132 sec. In general, during the processing of the WIDER Face data set, the best performance for MTCNN, PyramidBox and DSFD is achieved using the GPUs RTX 2060 and TITAN, and the CPUs i9-8650HK and Xeon E5, respectively. This indicates that on CPUs the detection speed is determined by the base frequency, number of cores and bus speed. Therefore, the CPU i9-8650HK, with high base frequency and bus speed, has a better performance in comparison to the CPU Xeon E5, with low base frequency and bus speed, regardless the high memory of the test computer and the CPU cache. In the case of GPUs, it is observed that the architecture determines the detection speed. Thus, RTX GPUs with Turing architecture performs better than GPUs with Pascal and Keppler architectures despite that these GPUs have a large number of cores, memory video or memory bandwidth. Furthermore, GPUs with a large number of cores, video memory and clock frequency perform face detection in less time.

#### 5.1.2. Results on the UFDD Data Set

**Figure 6:**
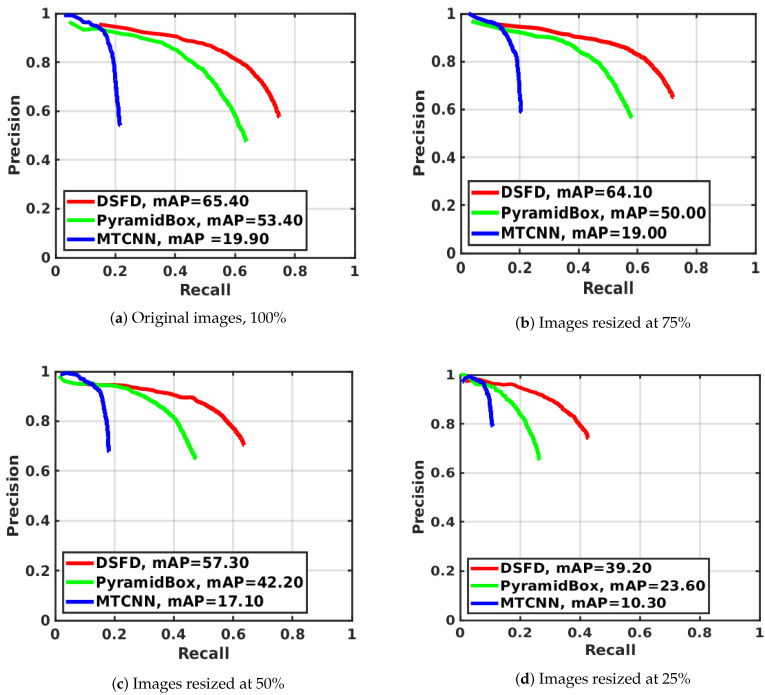
Precision-Recall curves on the UFDD Face data set for the MTCNN, PyramidBox and DSFD face detection methods using four different image resolutions.

**Figure 7:**
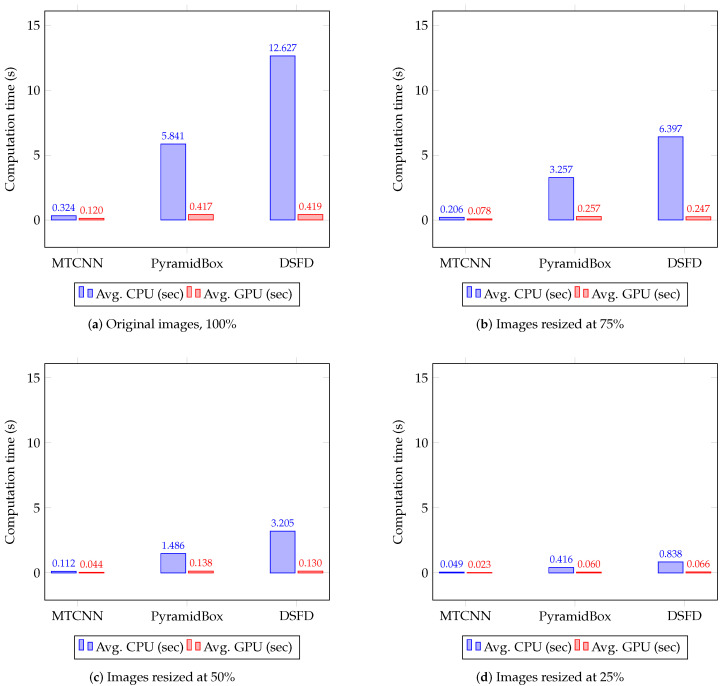
Average CPU and GPU computation time (s) on the UFDD Face data set for the MTCNN, PyramidBox and DSFD face detection methods using four different image resolutions.

Table 7 shows the mAP values, the F1 scores and the speed obtained for the evaluated face detectors using the considered image sizes, CPUs and GPUs. Figure 6 depicts the precision–recall curves of detectors, and Figure 7 presents the average processing time on CPUs and GPUs. In both figures, results are summarized by the analysed image resolutions. Table 8 reports the Impv of the mAP, the F1 score and the speed of the face detectors with different image resolutions against the results using full size images.

Consistent with the results obtained on the WIDER Face data set, MTCNN is the fastest face detector, while DSFD is the most accurate (see Table 7, Figure 6 and Figure 7). The mAP values range from 10.30% to 65.40%, whereas the F1 score values vary from 0.123 to 0.724.

Regarding the computation time (see Table 7), the best detection performance is achieved with the CPU i9-8650HK and the GPU RTX 2060, followed by the GPU TITAN Xp. Similar to the WIDER Face data set, on CPUs, it is observed that the base frequency, number of cores and bus speed determined the computational time during face detection. In contrast, the most relevant characteristics of GPUs are the architecture, number of cores, video memory and clock frequency. Furthermore, the use of GPUs speeds up face detectors in comparison to CPUs, reducing the processing time of complex detectors significantly. In particular, MTCNN shows an improvement in detection speed using GPUs between 52.68% and 62.87%, while DSFD (more computationally demanding) achieved a reduction in processing times between 92.16% and 96.68% using GPUs.

Moreover, the use of resized images speeds up face detection at the cost of a decrease in mAP and F1 scores. The smaller the input image size is, the higher reduction in mAP and F1 score are in comparison to the values achieved using the original images. Hence, the maximum improvement of the detection speed and decrease of the mAP is observed using CPUs and GPUs to process images resized to 25% of the original image size (see Table 8). In this case, the use of GPUs leads to the fastest face detection: MTCNN performed the detection in 0.023 sec with mAP of 10.30% and F1 score of 0.123; PyramidBox carried out detection in 0.060 sec with mAP of 23.60% and F1 score of 0.248 and DSFD achieved detection in 0.066 sec with mAP of 39.20% and F1 score of 0.419. This corresponds to an Impv in speed of 80.93% for MTCNN, 84.22% for PyramidBox and 84.31% for DSFD, and a reduction in mAP values of −48.24% for MTCNN, −55.81% for PyramidBox and −40.06% for DSFD. Similar reductions are observed for the F1 scores of the three face detectors.

The best speed–accuracy tradeoff is obtained on the UFDD data set using DSFD and GPUs with images reduced to 50% of the original size—average mAP of 57.30%, F1 score of 0.626 and processing time of 0.130 sec—or CPUs with images reduced to 25% of the original size—average mAP of 39.20%, F1 score of 0.419 and computation time of 0.698 sec. In the case of the best GPU set-up, the mAP and F1 score is improved considerably in comparison to the results attained with MTCNN and full size images—average mAP of 19.90%, F1 score of 0.236 and processing time of 0.120 sec—with a similar detection speed.

### 5.2. Performance Estimation Model

Here we assess Generalized Linear Models (GLMs) to estimate the processing time and the F1 score of face detectors. GLM [62] is a flexible generalization of ordinary linear regression models that assume that response variables, such as processing time and F1 scores, follow an error distribution which does not have to correspond to the Gaussian or normal distribution. The GLM parameters were estimated using the generalized least squares method. In this work, we compared GLMs assuming a normal distribution of variables against different data distributions, such as the Binomial Negative one. Furthermore, we assessed the improvement of GLMs through a logarithmic transformation of the response variables or a concatenation of explanatory variables. The logarithmic transformation is commonly employed in regression models to handle a non-linear relationship between the response and explanatory variables. The concatenation of explanatory variables is considered as a way to reduce the complexity of regression models which may improve the linear fit of the data.

#### 5.2.1. Processing Time Estimation

Table 9 reports the MAE, the RMSE and the MSE values for the regression models built to predict the computational time of face detectors based on the areas of images, face detection methods, image resized percentages, and hardware used to process images (see Section 3.5). Taking into account that the computational time may have an exponential tendency, we first applied a logarithmic transform to the detection speed before building GLMs (see rows 2 and 5). It was observed that using this data transformation improves the fit of regression models to predict the processing time, in particular, the performance of the baseline (model 1)—MAE of 1.438, MSER of 2.164 and MSE of 4.682—improved to MAE of 0.624, MSER of 1.851 and MSE of 3.425 (model 2). Furthermore, we compared models built with individual variables (see rows 1-3) against models trained using a concatenation of the categorical variables: method, resized and machine (see rows 4 and 5). Results show that the concatenation of variables improved the fit of regression models for estimating the computational time. In the best case (model 5), the combination of the concatenated variables along with speed logarithmic transform allows a significant improvement in performance—MAE of 0.113, MSER of 0.455 and MSE of 0.207—in comparison to the baseline (model 1).

#### 5.2.2. F1 Score Estimation

Table 10 shows the MAE, the RMSE and the MSE values for the regression models built to predict the F1 score of face detectors based on the areas of the images, face detection methods, and image resized percentages (see Section 3.5). Recall that the hardware is not considered as an explanatory variable since the detection has the same F1 score regardless of the CPUs or GPUs employed to process images. Besides, since the logarithmic function is defined for positive values larger than zero, and the F1 metric ranges between 0 and 1, it is not feasible to use logarithmic transformation in this case. Hence, we compared the performance of GLMs built with individual variables assuming a normal and a Binomial Negative distribution, models 1 and 2, respectively, against a model trained with a concatenation of the categorical variables: method and resized (see model 3). Results show that there is not a significant difference between the assessed models for F1 score estimation, having a slightly better performance the model built with a normal distribution and the concatenated variables—MAE of 0.370, MSER of 0.417 and MSE of 0.174.

## 6. Conclusions and Future Work

Deep learning approaches based on CNNs have proven to be highly effective for automated face detection achieving remarkable accuracy. Forensic face recognition, like CSEM detection systems, remains a more difficult task because it must be able to handle images captured under non-ideal conditions and meet stringent time constraints. These deep learning models, however, tend to be very computationally demanding, and some of them may not be appropriate for CSEM-like applications.

In this work, we present a comparison of the speed and the accuracy of three popular face detectors based on deep learning—MTCNN, PyramidBox, and DSFD—on five Intel CPUs—i5-3450, i7-4790K, i7-8650U, i9-8950HK, and Xeon E5—and seven Nvidia GPUs cards—Tesla K40, TITAN Xp, GTX 1050Ti, GTX 1060, GTX 1070, RTX 2060, and RTX 2070—by analyzing images reduced to three different images sizes from two data sets—WIDER Face and UFDD.

Results confirm that the use of resized images speeds up the face detection stage but reduces the accuracy. We found that the speed-up achieved by using resizing images and GPUs depends on the complexity of the face detector used. Thus, sophisticated detectors have a substantial improvement in processing times. It turns out that the best speed–accuracy tradeoff is yielded by applying the DSFD detector to images resized to 50% of the original size in GPUs and images resized to 25% of the original size in CPUs. Moreover, the best performances were obtained with CPU i9-8950HK and GPU RTX 2060.

Considering this tradeoff between speed and accuracy, we train a model capable of predicting for new images the performance of several face detectors with various resized images in a specified hardware. Experimental results with multiple linear regression models are able to predict the face detection performance with a MAE of 0.113.

The proposed models are expected to help end-users as forensic investigators to select the most appropriate hardware for applications where face detection is required, such as face recognition or child detection in CSEM. Additionally, the prediction model will guide the forensic practitioners to choose the best parameters (detection method and image resolution) for face detection considering the available computational resources.

Building more complex prediction models becomes part of our future work. Our aim is to also analyze the features that have the most influence on the model performance.

## Figures and Tables

**Table 1:** Face detection performance on the WIDER Face data set [36].

Method	Year	mAP per Category			Speed (FPS)	GPU	Code
		Easy(%)	Medium(%)	Hard(%)			
MTCNN [22]	2016	85.1	82.0	60.7	99.0	TITAN Black	Yes
S3DF [24]	2017	93.7	92.4	85.2	36.0	TITAN X	Yes
FANet [23]	2017	95.6	94.7	89.5	35.6	GTX 1080 ti	Yes
PyramidBox [25]	2018	96.1	95.0	89.5	—	—	Yes
DSFD [26]	2018	96.6	95.7	90.4	—	—	Yes
AInnoFace [27]	2019	96.5	95.7	91.2	—	—	No

**Table 2:** Events in the WIDER Face data set grouped by level of face detection difficulty [36].

Difficulty	Real World Events
Easy	Gymnastics, Handshaking, Waiter Waitress, Press Conference, Worker Laborer, Parachutist Paratrooper, Sports, Coach Trainer, Meeting, Aerobics, Row Boat, Dancing, Swimming, Family Group, Balloonist, Dresses, Couple, Jockey, Tennis, Spa, Surgeons.
Medium	Stock Market, Hockey, Students Schoolkids, Ice Skating, Greeting, Football, Running, People Driving Car, Soldier Drilling, Photographers, Sports Fan, Group, Celebration/Party, Soccer, Interview, Raid, Baseball, Soldier Patrol, Angler, Rescue.
Hard	Traffic, Festival, Parade, Demonstration, Ceremony, People Marching, Basketball, Shoppers, Matador Bullfighter, Car Accident, Election Campaign, Concerts, Award Ceremony, Picnic, Riot, Funeral, Cheering, Soldier Firing, Car Racing, Voter.

**Table 3:** Evaluated CPUs specification details. CPUs were installed in desktop${}^{+}$ computer, laptop${}^{†}$ or tablet surface pro${}^{*}$.

CPU	Base Frequency	Cores	Cache	Bus Speed	Memory
Intel i5-3450${}^{+}$	3.10 GHz	4	6 MB	5 GT/s	8 GB
Intel i7-8650U${}^{*}$	1.90 GHz	4	8 MB	4 GT/s	16 GB
Intel i7-4790K${}^{+}$	4.00 GHz	4	8 MB	5 GT/s	32 GB
Intel i9-8950HK${}^{†}$	2.90 GHz	6	12 MB	8 GT/s	32 GB
Intel Xeon E5-2630${}^{+}$	2.40 GHz	6	15 MB	7.2 GT/s	128 GB

**Table 4:** Evaluated GPUs specification details. GPUs were installed in desktop${}^{+}$ or laptop${}^{†}$ computers.

GPU	Arch.	Cores	Video	Memory	Clock	Memory
			Memory	Bandwidth	Frequency	
Tesla K40c${}^{+}$	Kepler	2880	12 GB	288 GB/s	745 MHz	128 GB
TITAN Xp${}^{+}$	Pascal	3840	12 GB	547.7 GB/s	1404 MHz	128 GB
GTX 1050 Ti${}^{+}$	Pascal	768	4 GB	112 GB/s	1290 MHz	32 GB
GTX 1060${}^{+}$	Pascal	1280	6 GB	162 GB/s	1506 MHz	32 GB
GTX 1070${}^{+}$	Pascal	1920	8 GB	256 GB/s	1506 MHz	32 GB
RTX 2060${}^{†}$	Turing	1920	6 GB	336 GB/s	1365 MHz	16 GB
RTX 2070${}^{†}$	Turing	2304	6 GB	448 GB/s	1410 MHz	16 GB

**Table 5:** Speed and accuracy (mAP and F1 score) tradeoff results on the WIDER Face data set for MTCNN, PyramidBox and DSFD face detection methods using four image resolutions, and different CPUs/GPUs configurations. The best mAP, F1 score and speed values per image size and face detector are highlighted in bold. Higher mAP and F1 score with lower speed values mean a better performance.

	Method	Full Size Image (100%)			Resized Image to 75%			Resized Image to 50%			Resized Image to 25%		
Metric		MTCNN	PyramidBox	DSFD	MTCNN	PyramidBox	DSFD	MTCNN	PyramidBox	DSFD	MTCNN	PyramidBox	DSFD
Avg. mAP (%)		56.10	92.47	**94.20**	56.40	92.57	**94.73**	54.07	89.57	**93.77**	45.67	72.40	**86.33**
Avg. F1 score		0.505	0.881	**0.917**	0.505	0.863	**0.914**	0.478	0.791	**0.883**	0.392	0.566	**0.743**
CPU i5-3450 (s)		0.333	6.749	18.211	0.203	3.766	10.017	0.113	1.689	4.429	0.051	0.448	1.053
CPU i7-8650U (s)		0.442	8.182	17.943	0.268	4.648	10.152	0.142	2.110	4.767	0.061	0.571	1.291
CPU i7-4790K (s)		0.225	5.784	11.872	0.135	3.231	6.170	**0.071**	1.418	2.699	0.030	0.375	0.581
CPU i9-8950HK (s)		**0.200**	**4.253**	10.036	**0.134**	**2.378**	5.396	0.078	**1.059**	2.335	**0.027**	**0.276**	**0.565**
CPU Xeon E5 (s)		0.573	4.658	**9.585**	0.367	2.810	**3.925**	0.219	1.414	**2.258**	0.101	0.500	0.884
Tesla K40c (s)		0.200	0.718	0.829	0.126	0.443	0.500	0.069	0.244	0.268	0.035	0.120	0.132
TITAN Xp (s)		0.141	**0.206**	0.278	0.091	**0.140**	0.181	0.054	**0.093**	0.110	0.031	0.055	0.066
GTX 1050 Ti (s)		0.116	0.649	0.648	0.073	0.355	0.371	0.042	0.180	0.171	**0.020**	0.057	0.078
GTX 1060 (s)		0.114	0.359	0.363	0.076	0.225	0.218	0.041	0.117	0.108	0.021	0.050	0.050
GTX 1070 (s)		0.123	0.268	0.320	0.079	0.169	0.201	0.046	0.095	0.111	0.023	0.046	0.063
RTX 2060 (s)		**0.112**	0.492	**0.260**	**0.070**	0.279	**0.169**	**0.040**	0.129	**0.076**	**0.020**	**0.045**	**0.042**
RTX 2070 (s)		0.119	0.493	0.276	0.075	0.271	0.183	0.043	0.129	0.077	**0.020**	0.046	0.051
Impv. GPU vs. CPU (%)		62.70	92.32	96.86	61.95	92.01	96.35	61.54	90.83	96.01	55.51	86.19	92.14

**Table 6:** Improvement (Impv) in terms of accuracy (mAP and F1 score) and speed obtained with different image resolutions—75%, 50% and 25%—with respect to values computed for full size images—baseline—using MTCNN, PyramidBox and DSFD, and different CPUs/GPUs configurations on the WIDER Face data set. The best Impv per image size and face detector is highlighted in bold. Higher Impv values indicate a better performance.

	Method	Img. 75% vs. Img. 100%			Img. 50% vs. Img. 100%			Img. 25% vs. Img. 100%		
Impv (%)		MTCNN	PyramidBox	DSFD	MTCNN	PyramidBox	DSFD	MTCNN	PyramidBox	DSFD
Avg. mAP		0.53	0.11	**0.57**	−3.62	−3.14	**−0.46**	−18.60	−21.70	**−8.35**
Avg. F1 score		**−0.06**	−2.05	−0.30	−5.36	−10.22	**−3.71**	−22.35	−35.72	**−18.91**
Avg. Speed CPUs		37.42	43.06	**48.35**	65.03	73.88	**75.91**	85.24	92.53	**93.46**
Avg. Speed GPUs		36.22	**39.81**	37.70	63.64	67.51	**68.61**	81.81	**85.45**	82.92

**Table 7:** Speed and accuracy (mAP and F1 score) tradeoff results on the UFDD data set for the MTCNN, PyramidBox and DSFD face detection methods using four different image resolutions, and CPUs/GPUs configurations. The best mAP, F1 score and speed values per image size and face detector are highlighted in bold. Higher mAP and F1 score with lower speed values mean a better performance.

	Method	Full Size Image (100%)			Resized Image to 75%			Resized Image to 50%			Resized Image to 25%		
Metric		MTCNN	PyramidBox	DSFD	MTCNN	PyramidBox	DSFD	MTCNN	PyramidBox	DSFD	MTCNN	PyramidBox	DSFD
Avg. mAP (%)		19.90	53.40	**65.40**	19.0	50.0	**64.10**	17.10	42.20	**57.30**	10.30	23.60	**39.20**
Avg. F1 score		0.236	0.576	**0.724**	0.224	0.546	**0.705**	0.199	0.451	**0.626**	0.123	0.248	**0.419**
CPU i5-3450 (s)		0.311	6.525	17.585	0.191	3.655	9.727	0.103	1.629	4.287	0.045	0.438	1.016
CPU i7-8650U (s)		0.407	7.991	17.077	0.248	4.467	11.002	0.128	2.035	4.773	0.053	0.551	1.263
CPU i7-4790K (s)		0.206	5.584	11.253	**0.124**	3.099	6.417	0.064	1.390	2.557	**0.027**	0.365	0.550
CPU i9-8950HK (s)		**0.184**	4.598	**9.425**	0.119	**2.354**	**0.376**	**0.066**	**1.023**	2.253	0.028	**0.267**	**0.525**
CPU Xeon E5(s)		0.510	**4.509**	7.795	0.347	2.711	4.462	0.199	1.351	**2.154**	0.091	0.459	0.835
Tesla K40c (s)		0.179	0.676	0.820	0.116	0.424	0.477	0.061	0.237	0.259	0.034	0.117	0.127
TITAN Xp (s)		0.130	**0.196**	0.276	0.086	**0.136**	0.179	0.050	**0.092**	0.110	0.030	0.056	0.068
GTX 1050 Ti (s)		0.105	0.608	0.636	0.066	0.347	0.359	**0.037**	0.184	0.170	**0.017**	0.063	0.067
GTX 1060 (s)		0.103	0.337	0.350	0.066	0.215	0.210	0.038	0.113	0.107	0.019	0.049	0.050
GTX 1070 (s)		0.111	0.249	0.311	0.073	0.162	0.196	0.042	**0.092**	0.110	0.022	0.045	0.063
RTX 2060 (s)		**0.102**	0.428	**0.252**	**0.065**	0.259	0.162	**0.037**	0.122	**0.076**	0.018	**0.045**	**0.041**
RTX 2070 (s)		0.111	0.426	0.287	0.072	0.253	**0.143**	0.042	0.124	0.079	0.021	0.048	0.045
Impv. GPU vs. CPU (%)		62.87	92.86	96.68	62.19	92.12	96.14	60.86	90.74	95.94	52.68	85.49	92.16

**Table 8:** Improvement (Impv) in terms of accuracy (mAP and F1 score) and speed obtained with different image resolutions—75%, 50% and 25%—with respect to values computed for full size images—baseline—using the MTCNN, PyramidBox and DSFD and different CPUs/GPUs configurations on the UFDD data set. The best Impv per image size and face detector are highlighted in bold. Higher Impv values indicate a better performance.

	Method	Img. 75% vs. Img. 100%			Img. 50% vs. Img. 100%			Img. 25% vs. Img. 100%		
Impv (%)		MTCNN	PyramidBox	DSFD	MTCNN	PyramidBox	DSFD	MTCNN	PyramidBox	DSFD
Avg. mAP		−4.52	−6.37	**−1.99**	−14.07	−20.97	**−12.39**	−48.24	−55.81	**−40.06**
Avg. F1 score		−5.29	−5.19	**−2.66**	−15.77	−21.81	**−13.55**	−48.12	−56.97	**−42.21**
Avg. Speed CPUs		36.97	44.25	**52.40**	65.91	74.49	**74.68**	85.28	92.77	**93.13**
Avg. Speed GPUs		35.32	37.45	**40.43**	63.36	65.70	**68.36**	80.93	**84.22**	83.31

**Table 9:** Mean Absolute Error (MAE), Root Mean Squared Error (RMSE) and Mean Squared Error (MSE) of the regression models built to predict the computational time (speed) based on the area of images (area), face detectors (method), image resized percentage (resized), and hardware used to process images (machine). Lower values of MAE, RMSE and MSE mean better performance.

#	GLM Regression	Explanatory variables	Prediction	MAE	RMSE	MSE
1	Gaussian distribution (normal)	area, method, machine, resized	speed	1.438	2.164	4.682
2	Normal + logarithmic (log) speed	area, method, machine, resized	log (speed)	0.624	1.851	3.425
3	Binomial Negative distribution	area, method, machine, resized	speed	0.287	0.840	0.705
4	Normal + variables concatenation (concat)	area, concat (method, machine, resized)	speed	0.246	0.636	0.405
5	**Normal + variables concat + log speed**	area, concat (method, machine, resized)	log (speed)	**0.113**	**0.455**	**0.207**

**Table 10:** MAE, RMSE and MSE of the regression models built to predict the F1 score (F1Score) based on the area of images (area), face detectors (method), and image resized percentage (resized). Lower values of MAE, RMSE and MSE mean better performance.

#	GLM Regression	Explanatory variables	Prediction	MAE	RMSE	MSE
1	Gaussian distribution (normal)	area, method, resized	F1Score	0.371	0.418	0.175
3	Binomial Negative distribution	area, method, resized	F1Score	0.370	0.446	0.199
2	**Normal + variables concatenation (concat)**	area, concat (method, resized)	F1Score	**0.370**	**0.417**	**0.174**

## References

[1] Anda F., Lillis D., Kanta A., Becker B.A., Bou-Harb E., Le-Khac N.A., Scanlon M. Improving Borderline Adulthood Facial Age Estimation Through Ensemble Learning. Proceedings of the 14th International Conference on Availability, Reliability and Security (ARES ’19).

[2] Carriquiry A., Hofmann H., Tai X.H., VanderPlas S. (2019). Machine learning in forensic applications. Significance.

[3] Rughani P.H., Bhatt P. (2017). Machine learning forensics: a new branch of digital forensics. Int. J. Adv. Res. Comput. Sci..

[4] Nixon M., Aguado A. (2019). Feature Extraction and Image Processing for Computer Vision.

[5] Gangwar A., Fidalgo E., Alegre E., González-Castro V. Pornography and Child Sexual Abuse Detection in Image and Video: A Comparative Evaluation. Proceedings of the 8th International Conference on Imaging for Crime Detection and Prevention (ICDP).

[6] Saikia S., Fidalgo E., Alegre E., Fernández-Robles L. Object Detection for Crime Scene Evidence Analysis Using Deep Learning. Proceedings of the Image Analysis and Processing (ICIAP).

[7] Chaves D., Saikia S., Fernández-Robles L., Alegre E., Trujillo M. (2018). A Systematic Review on Object Localisation Methods in Images. Rev. Iberoam. Automática Inf. Ind..

[8] Saikia S., Fidalgo E., Alegre E., Fernández-Robles L. Query Based Object Retrieval Using Neural Codes. Proceedings of the International Joint Conference SOCO’17-CISIS’17-ICEUTE’17.

[9] Nadeem M.S., Franqueira V.N., Zhai X., Kurugollu F. (2019). A Survey of Deep Learning Solutions for Multimedia Visual Content Analysis. IEEE Access.

[10] Schroff F., Kalenichenko D., Philbin J. Facenet: A unified embedding for face recognition and clustering. Proceedings of the IEEE conference on computer vision and pattern recognition.

[11] Biswas R., González-Castro V., Fidalgo E., Chaves D. (2019). Boosting child abuse victim identification in Forensic Tools with hashing techniques. V Jorn. Nac. Investig. Ciberseguridad.

[12] García-Olalla O., Alegre E., Fernández-Robles L., Fidalgo E., Saikia S. (2018). Textile Retrieval Based on Image Content from CDC and Webcam Cameras in Indoor Environments. Sensors.

[13] Singh S., Prasad S. (2018). Techniques and Challenges of Face Recognition: A Critical Review. Procedia Comput. Sci..

[14] Zafeiriou S., Zhang C., Zhang Z. (2015). A survey on face detection in the wild: Past, present and future. Comput. Vis. Image Underst..

[15] Zhou Y., Liu D., Huang T. Survey of face detection on low-quality images. Proceedings of the 2018 13th IEEE International Conference on Automatic Face & Gesture Recognition (FG 2018).

[16] Kumar A., Kaur A., Kumar M. (2019). Face detection techniques: A review. Artif. Intel. Rev..

[17] Viola P., Jones M.J. (2004). Robust real-time face detection. Int. J. Comput. Vis..

[18] Bay H., Tuytelaars T., Van Gool L. Surf: Speeded up robust features. Proceedings of the European Conference on Computer Vision.

[19] Dalal N., Triggs B. Histograms of oriented gradients for human detection. Proceedings of the 2005 IEEE Computer Society Conference on Computer Vision and Pattern Recognition (CVPR’05).

[20] Ahonen T., Hadid A., Pietikainen M. (2006). Face description with local binary patterns: Application to face recognition. IEEE Trans. Pattern Anal. Mach. Intell..

[21] Zhu X., Ramanan D. Face detection, pose estimation, and landmark localization in the wild. Proceedings of the 2012 IEEE conference on computer vision and pattern recognition.

[22] Zhang K., Zhang Z., Li Z., Qiao Y. (2016). Joint Face Detection and Alignment Using Multitask Cascaded Convolutional Networks. IEEE Signal Process Lett..

[23] Zhang J., Wu X., Zhu J., Hoi S.C.H. (2017). Feature Agglomeration Networks for Single Stage Face Detection. arXiv.

[24] Zhang S., Zhu X., Lei Z., Shi H., Wang X., Li S.Z. S^3^FD: Single Shot Scale-Invariant Face Detector. Proceedings of the IEEE International Conference on Computer Vision (ICCV).

[25] Tang X., Du D.K., He Z., Liu J. PyramidBox: A Context-assisted Single Shot Face Detector. Proceedings of the European Conference on Computer Vision (ECCV).

[26] Li J., Wang Y., Wang C., Tai Y., Qian J., Yang J., Wang C., Li J., Huang F. DSFD: Dual Shot Face Detector. Proceedings of the IEEE/CVF Conference on Computer Vision and Pattern Recognition (CVPR).

[27] Zhang F., Fan X., Ai G., Song J., Qin Y., Wu J. (2019). Accurate Face Detection for High Performance. arXiv.

[28] Singh N.S., Hariharan S., Gupta M., Jain V., Chaudhary G., Cengiz Taplamacioglu M., Agarwal M.S. (2020). Facial Recognition Using Deep Learning. Advances in Data Sciences, Security and Applications.

[29] Swapna M., Sharma Y.K., Prasad B., Srujan Raju K., Senkerik R., Prasad Lanka S., Rajagopal V. (2020). A Survey on Face Recognition Using Convolutional Neural Network. Data Engineering and Communication Technology.

[30] Sayed A.R.E., Chakik A.E., Alabboud H., Yassine A. (2017). 3D face detection based on salient features extraction and skin colour detection using data mining. Imaging Sci. J..

[31] Abbasnejad I., Sridharan S., Nguyen D., Denman S., Fookes C., Lucey S. Using Synthetic Data to Improve Facial Expression Analysis with 3D Convolutional Networks. Proceedings of the 2017 IEEE International Conference on Computer Vision Workshops (ICCVW).

[32] Barbu A., Lay N., Gramajo G., Chellappa R., Theodoridis S. (2018). Chapter 6—Face detection with a 3D model. Academic Press Library in Signal Processing, Volume 6.

[33] Carlotta Olivetti E., Violante M., Vezzetti E., Marcolin F., Eynard B. (2020). Engagement Evaluation in a Virtual Learning Environment via Facial Expression Recognition and Self-Reports: A Preliminary Approach. Appl. Sci..

[34] Chaves D., Fidalgo E., Alegre E., Blanco P. Improving Speed-Accuracy Trade-off in Face Detectors for Forensic Tools by Image Resizing. Proceedings of the V Jornadas Nacionales de Investigación en Ciberseguridad (JNIC-2019).

[35] Chaves D., Fidalgo E., Alegre E., Jáñez-Martino F., Velasco-Mata J. CPU vs GPU performance of deep learning based face detectors using resized images in forensic applications. Proceedings of the 9th International Conference on Imaging for Crime Detection and Prevention (ICDP-2019).

[36] Yang S., Luo P., Loy C.C., Tang X. WIDER FACE: A Face Detection Benchmark. Proceedings of the IEEE Conference on Computer Vision and Pattern Recognition (CVPR).

[37] Nada H., Sindagi V.A., Zhang H., Patel V.M. Pushing the Limits of Unconstrained Face Detection: a Challenge Dataset and Baseline Results. Proceedings of the IEEE 9th International Conference on Biometrics Theory, Applications and Systems (BTAS).

[38] Everingham M., Gool L.V., Williams C.K.I., Winn J., Zisserma A. (2010). The Pascal Visual Object Classes (VOC) Challenge. Int. J. Comput. Vis..

[39] Grm K., Štruc V., Artiges A., Caron M., Ekenel H.K. (2017). Strengths and weaknesses of deep learning models for face recognition against image degradations. Iet Biometrics.

[40] Sawat D.D., Hegadi R.S. (2017). Unconstrained face detection: a deep learning and machine learning combined approach. CSI Trans. ICT.

[41] Masi I., Wu Y., Hassner T., Natarajan P. Deep face recognition: A survey. Proceedings of the 2018 31st SIBGRAPI Conference on Graphics, Patterns and Images (SIBGRAPI).

[42] Sun X., Wu P., Hoi S.C. (2018). Face detection using deep learning: An improved faster RCNN approach. Neurocomputing.

[43] Li C., Wang R., Li J., Fei L., Jain V., Patnaik S., Vlădicescu F.P., Sethi I.K. (2020). Face Detection Based on YOLOv3. Recent Trends in Intelligent Computing, Communication and Devices.

[44] You M., Han X., Xu Y., Li L. (2020). Systematic evaluation of deep face recognition methods. Neurocomputing.

[45] Wang M., Deng W. (2020). Deep face recognition with clustering based domain adaptation. Neurocomputing.

[46] Simonyan K., Zisserman A. (2014). Very deep convolutional networks for large-scale image recognition. arXiv.

[47] Lin T., Goyal P., Girshick R., He K., Dollár P. Focal Loss for Dense Object Detection. Proceedings of the 2017 IEEE International Conference on Computer Vision (ICCV).

[48] Zitnick C.L., Dollár P. Edge boxes: Locating object proposals from edges. Proceedings of the European Conference on Computer Vision.

[49] Zhang S., Wang X., Lei Z., Li S.Z. (2019). Faceboxes: A CPU real-time and accurate unconstrained face detector. Neurocomputing.

[50] Shi S., Wang Q., Xu P., Chu X. Benchmarking State-of-the-Art Deep Learning Software Tools. Proceedings of the 7th International Conference on Cloud Computing and Big Data.

[51] Nardelli R., Dall Z., Skevoulis S. Comparing TensorFlow Deep Learning Performance and Experiences Using CPUs via Local PCs and Cloud Solutions. Proceedings of the Advances in Information and Communication (FICC 2019).

[52] Demirović D., Skejić E., Šerifović-Trbalić A. Performance of Some Image Processing Algorithms in Tensorflow. Proceedings of the 2018 25th International Conference on Systems, Signals and Image Processing (IWSSIP).

[53] Huang B., Chen R., Zhou Q., Yu X. (2018). Eye landmarks detection via two-level cascaded CNNs with multi-task learning. Signal Process. Image Commun..

[54] Wang D., Yang J., Deng J., Liu Q. (2016). FaceHunter: A multi-task convolutional neural network based face detector. Signal Process. Image Commun..

[55] Ranjan R., Patel V.M., Chellappa R. (2019). HyperFace: A Deep Multi-Task Learning Framework for Face Detection, Landmark Localization, Pose Estimation, and Gender Recognition. IEEE Trans. Pattern Anal. Mach. Intell..

[56] Fang Z., Ren J., Marshall S., Zhao H., Wang Z., Huang K., Xiao B. (2020). Triple loss for hard face detection. Neurocomputing.

[57] Li Z., Tang X., Han J., Liu J., He R. (2019). PyramidBox++: High Performance Detector for Finding Tiny Face. arXiv.

[58] Deng J., Guo J., Zhou Y., Yu J., Kotsia I., Zafeiriou S. (2019). RetinaFace: Single-stage Dense Face Localisation in the Wild. arXiv.

[59] Liu W., Anguelov D., Erhan D., Szegedy C., Reed S., Fu C.Y., Berg A.C. SSD: Single Shot MultiBox Detector. Proceedings of the Computer Vision–ECCV 2016.

[60] Powers D.M. (2011). Evaluation: from Precision, Recall and F-measure to ROC, Informedness, Markedness and Correlation. J. Mach. Learn. Technol..

[61] Botchkarev A. (2019). A New Typology Design of Performance Metrics to Measure Errors in Machine Learning Regression Algorithms. Interdiscip. J. Inf. Knowl. Manag..

[62] McCullagh P., Nelder J. (2018). Generalized Linear Models.

